# The *RIP140* Gene Is a Transcriptional Target of E2F1

**DOI:** 10.1371/journal.pone.0035839

**Published:** 2012-05-18

**Authors:** Aurélie Docquier, Patrick Augereau, Marion Lapierre, Pierre-Olivier Harmand, Eric Badia, Jean-Sébastien Annicotte, Lluis Fajas, Vincent Cavaillès

**Affiliations:** 1 IRCM, Institut de Recherche en Cancérologie de Montpellier, and INSERM, U896, Montpellier, France; 2 Université Montpellier1, Montpellier, France; 3 CRLC Centre Régional de Lutte contre le Cancer Val d’Aurelle Paul Lamarque, Montpellier, France; Institut de Génomique Fonctionnelle de Lyon, France

## Abstract

RIP140 is a transcriptional coregulator involved in energy homeostasis and ovulation which is controlled at the transcriptional level by several nuclear receptors. We demonstrate here that RIP140 is a novel target gene of the E2F1 transcription factor. Bioinformatics analysis, gel shift assay, and chromatin immunoprecipitation demonstrate that the RIP140 promoter contains bona fide E2F response elements. In transiently transfected MCF-7 breast cancer cells, the RIP140 promoter is transactivated by overexpression of E2F1/DP1. Interestingly, RIP140 mRNA is finely regulated during cell cycle progression (5-fold increase at the G1/S and G2/M transitions). The positive regulation by E2F1 requires sequences located in the proximal region of the promoter (−73/+167), involves Sp1 transcription factors, and undergoes a negative feedback control by RIP140. Finally, we show that E2F1 participates in the induction of RIP140 expression during adipocyte differentiation. Altogether, this work identifies the RIP140 gene as a new transcriptional target of E2F1 which may explain some of the effect of E2F1 in both cancer and metabolic diseases.

## Introduction

RIP140 (Receptor Interacting Protein of 140 kDa also known as NRIP1) is a nuclear protein of 1158 amino acids, initially identified as a transcription cofactor of estrogen receptors which, despite its recruitment in the presence of agonist ligands, exhibits a strong transcriptional repressive activity of various nuclear receptors (for a review see [1]). The molecular mechanisms involved in this transrepression implicate several repressive domains within the RIP140 molecule and recruitment of different partners such as HDACs and CtBPs. Several post-translational modifications (such as acetylation, methylation and conjugation to SUMO peptides or vitamin B6) also play key roles in the regulation of RIP140 activity [2]-[3]. Interestingly, we previously demonstrated that RIP140 was involved in several transcriptional regulatory loops since its expression was increased upon estrogen or androgen stimulation, in breast [4] and prostate [5] cancer cells, respectively. Cloning of the human [6] and mouse [7] RIP140 genes showed that the overall organization was conserved and allowed identification of several cis-acting elements involved in transcriptional regulation by estrogens and dioxin [6] or by the nuclear receptor ERRα [7].

Molecular and cellular analyses together with *in vivo* approaches using knock-out mice have highlighted the role of RIP140 in various physiological and pathological processes [8]. For instance, this gene is required for a proper oocyte release during ovulation and involved in the regulation of fat accumulation and energy homeostasis in metabolic tissues. We recently reported that its expression is significantly decreased in basal-like breast cancers as compared to luminal ones [9]. We also demonstrated that RIP140 is involved in the control of cell proliferation and that it negatively regulated the activity of E2Fs [9].

The E2F family represents a class of transcription factors which regulate a broad spectrum of genes involved in major cellular processes such as DNA replication, apoptosis, differentiation and cell cycle [10]. E2F1, the founding member of the E2F family, has been shown to possess oncogenic properties and numerous evidences show that deregulation of E2F activity plays a key role in tumorigenesis [11]-[12]. In addition to its effect on cell cycle progression, E2F1 can also induce apoptosis through p53-dependent and -independent mechanisms [13] and data indicate that E2F1 behaves as a tumor suppressor *in vivo* [10]. More recently, a clear implication of E2F1 has been demonstrated in different metabolic processes including lipid and adipocyte metabolism or glucose homeostasis (for a review see [14]).

Eight E2F genes have been identified in mammals which encode proteins classified as transcriptional activators (E2F1–3) or repressors (E2F4–8) [15]. Most of the E2F family members exhibit structural conserved features such as a DNA-binding domain and hydrophobic heptad repeats which allow heterodimerization with DP proteins (DRTF1 polypeptides DP1, DP2 and DP3) through coil-coil interactions. In their C-terminus moiety, E2F1 to 5 exhibit a transactivation domain whose activity is tightly regulated by different post-translational modifications (such as phosphorylation and acetylation) and through the binding of pocket proteins (pRB, p107 and p130). E2F transcriptional activity is controlled by a plethora of factors [16]. The association with pocket proteins allows active repression through the recruitment of histone deacetylases and methyltransferases and is finely regulated by various members of the cyclin/cdk family [17].

The present study further deciphers the role of RIP140 in the E2F signaling pathway. Our data identify the RIP140 gene as a novel transcriptional target of E2F1 that might be involved in a wide range of pathological processes regulated by this transcription factor such as cancer or metabolic diseases.

## Materials and Methods

### Plasmids and Reagents

The pcDNA3-E2F1 and 4, pCMV-DP1, pCMV-Rb expression vectors were given by Dr C. Sardet (IGMM, Montpellier, France) and the pCMV-E2F2, 3, 5 and E2F6 expression vectors by Dr K. Helin (European Institute of Oncology, Milan, Italia). The reporter plasmids (CyclinE-luc and (E2F)_3_-TK-Luc) were obtained from Dr L. Fajas (IRCM, Montpellier). The pEFc-mycRIP140 expression vectors and the plasmid containing the RIP140 promoter (RIP900) have been described previously [6]. The pRL-CMVBis plasmid expressing Renilla luciferase (Stephan Vagner, Toulouse, France) was used to normalize transfection efficiency. The deletion of the Sp1 interaction domain in the pCDNA3 E2F1 sequence (from residues 109 to 121) [18] and the mutation in the DNA-binding domain (E132) [19] were done using the QuickChange XL kit (Stratagene). The same protocol was used to generate DP1 mutants in the pCMV expression vector (DP1 Δ107–126 and DP1 Δ205–277) corresponding respectively to the deletion of the DNA-binding [20] or E2F1 [21] interaction domains.

### Bioinformatics

Localization of human [6] and mouse [7] NRIP1 promoters (GenBank accession numbers AF127577 and AC145744 respectively) have been described previously. Potential transcription factor binding elements in the promoter region of both promoters were searched using Genomatix MatInspector Program (www.genomatix.de). Alignment of human and mouse NRIP1 promoter sequences and identification of the evolutionary conserved transcription factor binding elements were performed using Genomatix DiAlign Program (www.genomatix.de).

### Cell Culture

MCF-7 and HeLa human cancer cell lines were purchased from the American Type Culture Collection and routinely maintained in the laboratory as previously described [9]. For synchronization, 2×10^6^ cells HeLa cells were grown in 100 mm culture dishes to 60% of confluence in medium containing 0.5% FCS (serum starvation) and 2 mM HU (Boehringer Mannheim) was added during 24 hr to induce cell synchronization. When cells were at 80% of confluence, medium was removed to release the block. At each time point, cells were washed, trypsinized and total RNA was extracted. Cell cycle analysis was performed using a FACs-Vatange flow cytometer (Becton-Dickinson, San Jose, CA). For each sample, 2.10^4^ events were collected and the CellQuest software was used to analyze the list-mode data. ModFit software was used to determine the percentage of each G_0_/G_1_, S and G_2_/M phase in the population.

### Gel-Shift Assay

Gel shift assays were performed as previously described [6]. Sequences of sense strand oligonucleotides are given in Table S1. Where indicated, anti-E2F1 antibody (Ab) was added in the incubation mixtures, 5 min before the radioactive probe. Complexes were separated 15 min. later on non denaturing 4.5% polyacrylamide gel electrophoresis (acrylamide/bisacrylamide, 29∶1) in 0.25× Tris borate-EDTA at 150 V for 2 h, gel-fixed in 40% methanol/10% acetic acid, dried, and exposed overnight.

### ChIP Analysis

For ChIP analysis, MCF-7 cells (70% confluent) were cross-linked with 3,7% formaldehyde during 10 min at 37°C. The ChampionChIP One-Day Kit (SABiosciences) was then used according to the manufacturer’s recommendations. Immunoprecipitations were performed using the KH95 (sc-251 SantaCruz), C-20 (sc-866 Santa-Cruz) or 2656C6a (sc-81370 Santa-Cruz) antibodies against E2F1, E2F4 and RIP140 respectively. It should be noted that although the anti-E2Fs antibody were given by the supplier as being specific, we could not eliminate with certainty the possibility that they may cross-react with other E2Fs. As a negative control, we performed IP with no antibody (in our hands, such IP controls gave the same background as an isotype-matched mAb). Quantitative PCR was then performed using the Power SYBR Green PCR master mix, on an Applied Biosystems 7300 thermal cycler with 2 µl of material per point. Primers flanking the E2F site of the RIP140 and cyclin A2 promoters are given in Table S1. The input DNA fraction corresponded to 1% of the immunoprecipitation.

### Transient Transfection and Luciferase Assays

MCF-7 cells were plated in 96-well plates (20,000 cells per well). 24 h later, plasmid tests containing firefly luciferase reporter gene (25 ng), pRLCMVBis (25 ng per well) used as internal standard, and 25 ng each of different factors (E2F1, 2, 3, 4, 5, 6 and DP1) and 200 ng each of either RIP140, pRb, p130 proteins to a total of 0.25 µg total DNA per well, were cotranfected using Jet-PEI. 48 h after transfection, cells were lysed and firefly luciferase values were measured and normalized with the Renilla luciferase activity; all triplicate point values were expressed as mean + SD.

### Western-blot Analysis

Expression plasmids for E2F1, 2, 3, 4 or DP1 were transfected in MCF-7 cells using JetPEI. After cell lysis in 25 mM Tris-HCl, pH7.8, 2 mM EDTA, 2 mM DTT, 10% Glycerol, 1% Triton, supplemented with protease inhibitors, whole cell extracts were diluted in Laemmli sample buffer 2X and resolved by SDS–PAGE. Western blotting detection was performed using primary antibodies against E2Fs (sc-251, sc-9967, sc-56665, sc-1082 for E2F1, 2, 3, 4 respectively and sc-53642 for DP1 from Santa-Cruz Biotechnology). To check for gel loading, we used the anti-actin (A2066 from Sigma) or anti-TBP (MA1-21516 from ThermoScientific) antibodies.

### RNA Extraction and Quantitative PCR

Total RNA was extracted from cells as previously described [9]. PCR were carried out in a final volume of 10 µl using 0.5 µl of each primer (shown in Table S1) (10 µM), 2 µl of the supplied enzyme mix, 4 µl of H2O, and 3 µl of the template diluted at 1∶20. After a 10 min preincubation at 95°C, runs corresponded to 45 cycles of 15 s each at 95°C, 7 s at 57°C and 15 s at 72°C. Melting-curves of the PCR products were analyzed using the LightCycler software system to exclude amplification of unspecific products. Results were corrected for RS9 mRNA levels (reference gene) and normalized to a calibrator sample.

### Adipocyte Differentiation Experiments

E2F1^−/−^ and wild-type mouse embryonic fibroblasts (MEFs) were isolated from 13.5 days-old embryos in compliance with the French guidelines for experimental animal studies (agreement B34-172-27). Three different MEF cultures were obtained from E2F1 wild-type and knock-out embryos and grown in F12-DMEM supplemented with 10% fetal calf serum and 1.5% HEPES. For adipocyte differentiation experiments, MEFs (at passage 3) were seeded in 6-well plates. Two days after confluence, differentiation was induced by treating cells for 2 days with a cocktail containing 0.5 mM IBMX (3-isobutyl-1-methylxanthine), 10 µg/mL insulin, 1 µM dexamethasone and 1 µM BRL49653. Every two days, medium was changed with F12-DMEM, 10% serum, 1.5% HEPES, 10 µg/mL insulin and 1 µM BRL49653. Differentiated cells were visualized with Oil Red O staining (Sigma). Nuclear protein extracts were prepared using the NE-PER kit (Thermo Scientific) and 20 µg of nuclear cell extracts were analyzed by Western blotting using primary antibodies against E2F1 (sc-193 from Santa-Cruz Biotechnology) and RIP140 (H300 from Santa-Cruz Biotechnology). Total RNA was extracted using the Quick-RNA Miniprep (Zymo Research) and 1 µg was analyzed for RIP140 mRNA levels by real-time quantitative Polymerase Chain Reaction on an Applied Biosystems 7300 thermal circler. Results were corrected for RS9 mRNA levels used as a reference gene.

### Statistics

Statistical analysis was performed using the Student t test except for Figure S1 where we used the paired t-test (*p<0.05, **p<0.005 and ***p<0.0001).

## Results

### Identification of *Bona Fide* E2F Binding Sites in the RIP140 Promoter

**Figure 1:**
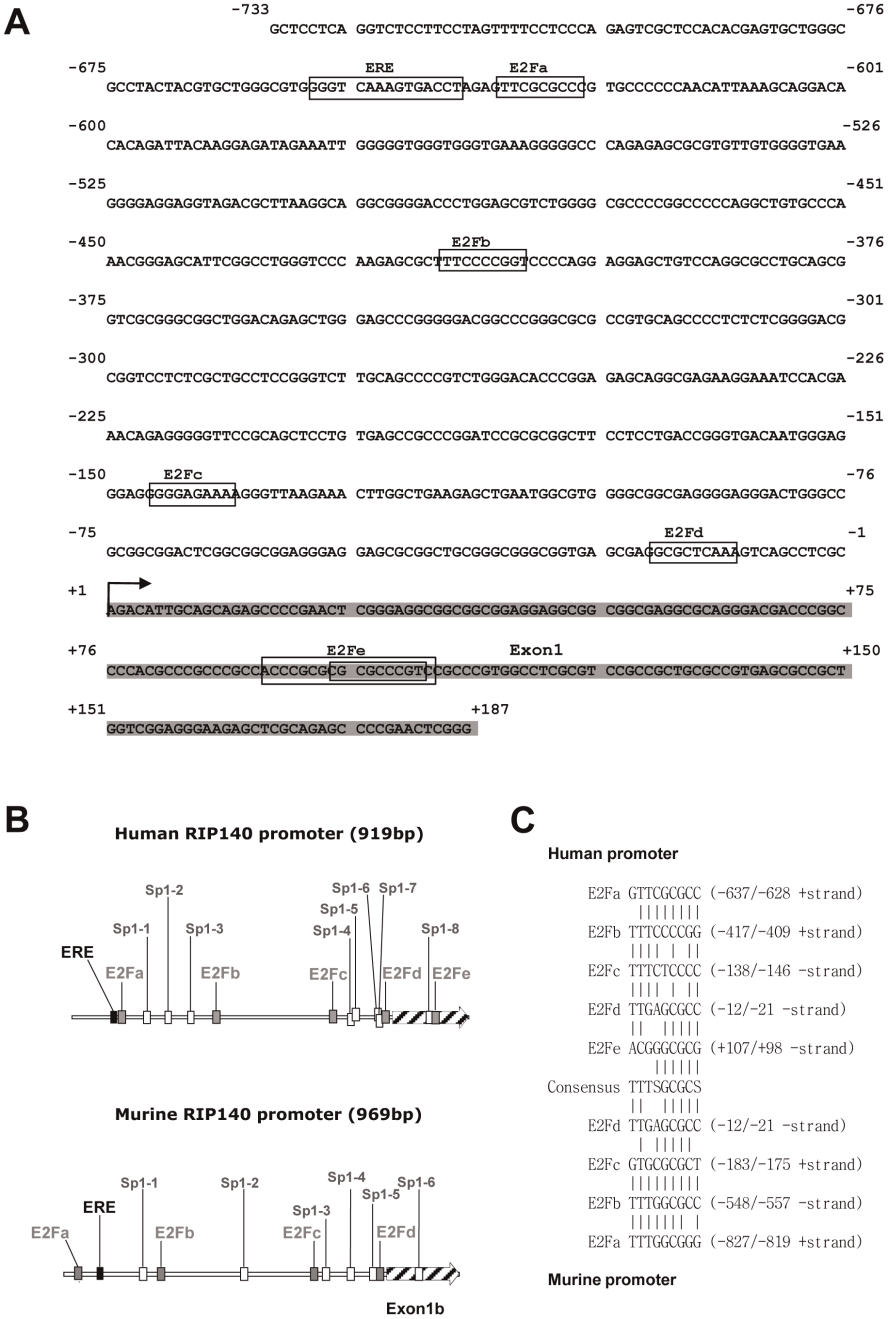
Localization of putative E2F binding sites in the RIP140 promoter. (**A**) Sequence of the human RIP140 gene promoter region. (**B**) Schematic representation of human and mouse RIP140 promoters. The human and mouse promoters exhibit 4 to 5 potential E2F binding sites (grey square) with a conserved distribution. Bioinformatics analysis also identified Sp1 binding sites (white square) in the murine (6 Sp1 sites) and in the human promoters (8 sites in two clusters). (**C**) Alignment of the different putative E2F binding sites found in the human and mouse promoters with the consensus sequence.

We previously reported the cloning and characterization of the human RIP140 gene promoter with the identification of various response elements [6]. Upon close inspection of the proximal promoter region, we mapped several putative E2F binding sites resembling the consensus sequence TTTSGCGCS. In the human RIP140 promoter, these sites were located at −637, −417, −146, −21 and +98 bp from the 5′ extremity of the cDNA (Figure 1A and B). These sites spread into two clusters with sites a and b being in the distal part of the promoter and sites c, d and e around the transcription start site, all being flanked by putative Sp1 sites. It should be noted that the E2Fa site exhibited the sequence which is the closest to the consensus motif with only one nucleotide change and that the E2Fe site was in fact a composite site with three different possibilities to bind the E2F/DP heterodimers (Figure 1A). Interestingly, the murine RIP140 promoter contains four putative E2F binding sites, the E2Fd site being perfectly conserved in term of position and sequence as compared to the human promoter (Figure 1 B and C).

**Figure 2:**
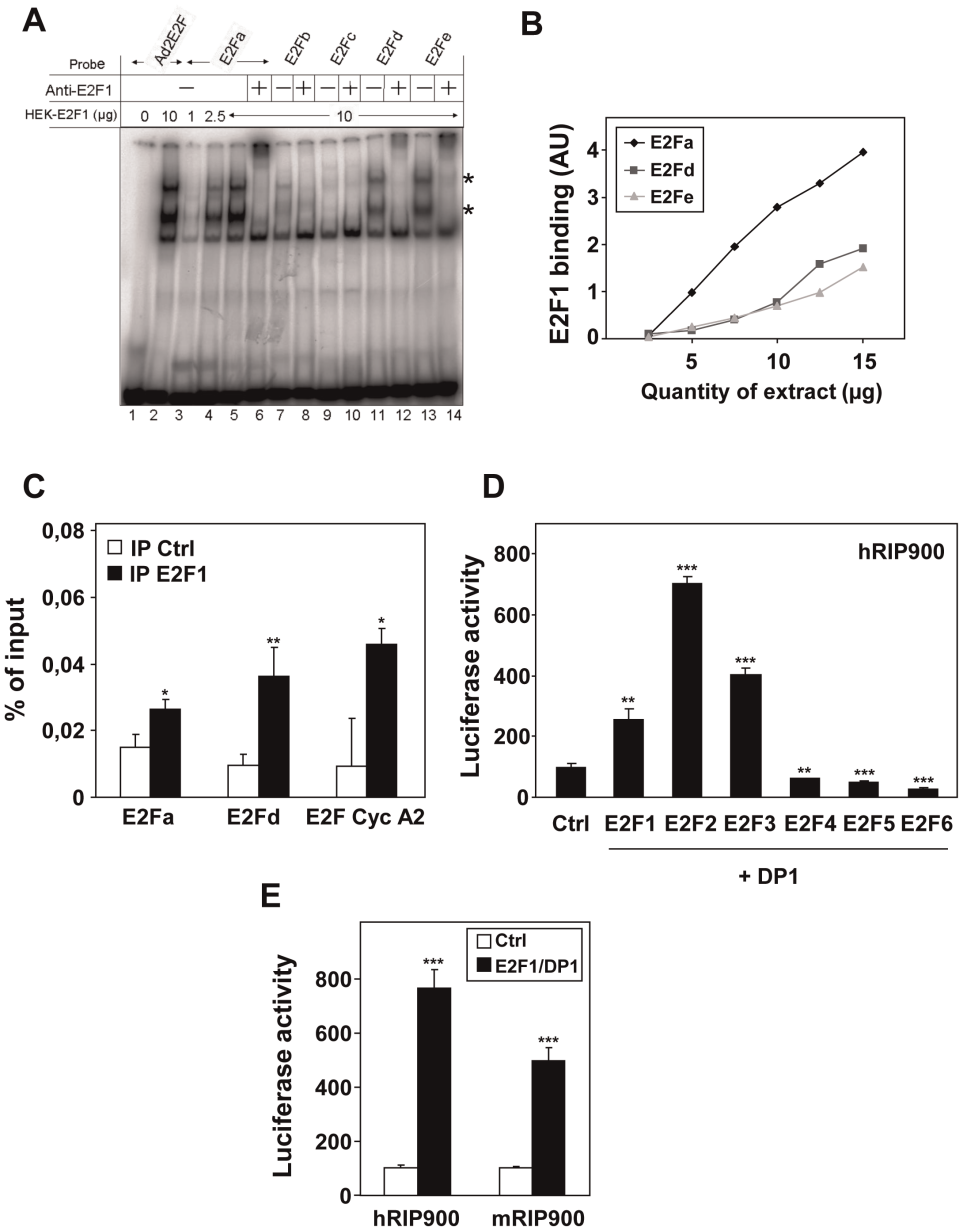
Analysis of E2F1 binding on the RIP140 promoter. (**A**) Electromobility shift assay was used to analyze E2F1/DP1 binding on the adenoviral E2F response element (Ad2E2F) or on the E2Fa, b, c, d and e sites of the RIP140 promoter (viewed in Figure 1). Asterisks indicate the retarded bands which contain E2F1. (**B**) Dose response experiment using increasing amounts of E2F1/DP1 on E2Fa, d and e binding sites. (**C**) ChIP experiments using immunoprecipitation (IP) of E2F1 on the E2Fa and E2Fd sites of the human RIP140 promoter. The cyclin A2 promoter was used as a positive control. Data are expressed as percent of the PCR signal obtained with the amount of chromatin used for IP (input). Negative control of IP was performed without any antibody which, in our hands, gave the same background as an isotype-matched mAb. Statistical analysis was performed using the Student t test (*p<0.05 and **p<0.005). (**D**) Human RIP900-luc (25 ng), E2F1 to 6 (25 ng) and DP1 (25 ng) were overexpressed in the indicated combinations. Relative luciferase activity was normalized with renilla luciferase activity as described in *Materials and Methods.* The values shown are from a representative experiment out of 3 data sets. They are expressed as a percentage of the activity obtained with control and are the mean (±SD) of triplicate. Statistical analysis performed using the Student t test (**p<0.005 and ***p<0.0001). (**E**) MCF-7 cells were transiently transfected with the human and murine RIP140 promoter reporter plasmids (25 ng) together with expression vectors for E2F1, DP1 (25 ng each). Results were expressed as described in panel D and a representative experiment is shown (n = 7).

We then checked the ability of the five different sites from the human promoter to act as *bona fide* E2F binding sites. Using gel shift experiments, we demonstrated that E2F1 strongly interacts with oligonucleotides encompassing some of the putative binding sites (*i.e.* E2Fa, E2Fd and E2Fe). As shown in Figure 2A, specific retarded bands (marked with an asterisk) were obtained when the labeled target sequence was incubated with increasing amounts of whole cell extract prepared from cells overexpressing E2F1 and DP1. The effect was comparable to that obtained with a consensus E2F binding site from the adenovirus gene (Ad2E2F) and these retarded bands were all shifted with an anti-E2F1 antibody. In these experiments, we found that the apparent binding affinities of E2F1/DP1 for the different motifs ranked as follows E2Fa>E2Fd = E2Fe (Figure 2B). No significant binding was observed for E2Fb and c (Figure 2A) and point mutations known to abolish E2F binding indeed hampered the interaction both in direct gel shift assays and in competition experiments (data not shown).

In order to demonstrate that the interaction of E2F1/DP1 also occurred in intact cells, we performed chromatin immunoprecipitation experiments. As shown in Figure 2C, the regions of the RIP140 promoter encompassing the E2Fa or E2Fd binding sites were PCR amplified at higher levels after immunoprecipitation with the anti-E2F1 antibody as compared to background levels of amplification of the same region after immunoprecipitation in the absence of the relevant antibody. The signal obtained after amplification of the RIP140 promoter was comparable to that obtained with the well-known E2F-target gene cyclin A2. Altogether, these data strongly suggested that the RIP140 gene could be an E2F target.

### Regulation of the RIP140 Promoter by E2Fs

To demonstrate a transcriptional regulation of the RIP140 promoter by E2Fs, we transiently transfected MCF-7 breast cancer cells with the RIP900 reporter construct containing the 900 bp of the RIP140 promoter fused to the luciferase coding sequence. This construct (which encompassed the five E2F binding sites that we identified) was cotransfected with expression vectors encoding the different members of the E2F family (Figure 2D and Figure S1). When cotransfected with DP1, we found that E2F1, E2F2 and E2F3 strongly increased luciferase activity from the RIP900 reporter (2.5 to 7-fold). Western-blot analysis confirmed the overexpression of the different E2Fs and that of DP1 (see Figure S3B and C). As expected increasing concentrations of expression vectors for E2F1 and DP1 produced a clear dose-dependent induction of luciferase activity on both the human and murine RIP140 reporters (data not shown). By contrast, E2F4, E2F5 and E2F6 were not able to transactivate the RIP140 promoter as expected since these factors are considered as transcriptional repressors. As shown in Figure 2D, we observed a significant decrease (1.6 to 3-fold; p<0.001) in luciferase activity when E2F4–6 were overexpressed.

### Regulation of Endogenous RIP140 Expression

**Figure 3:**
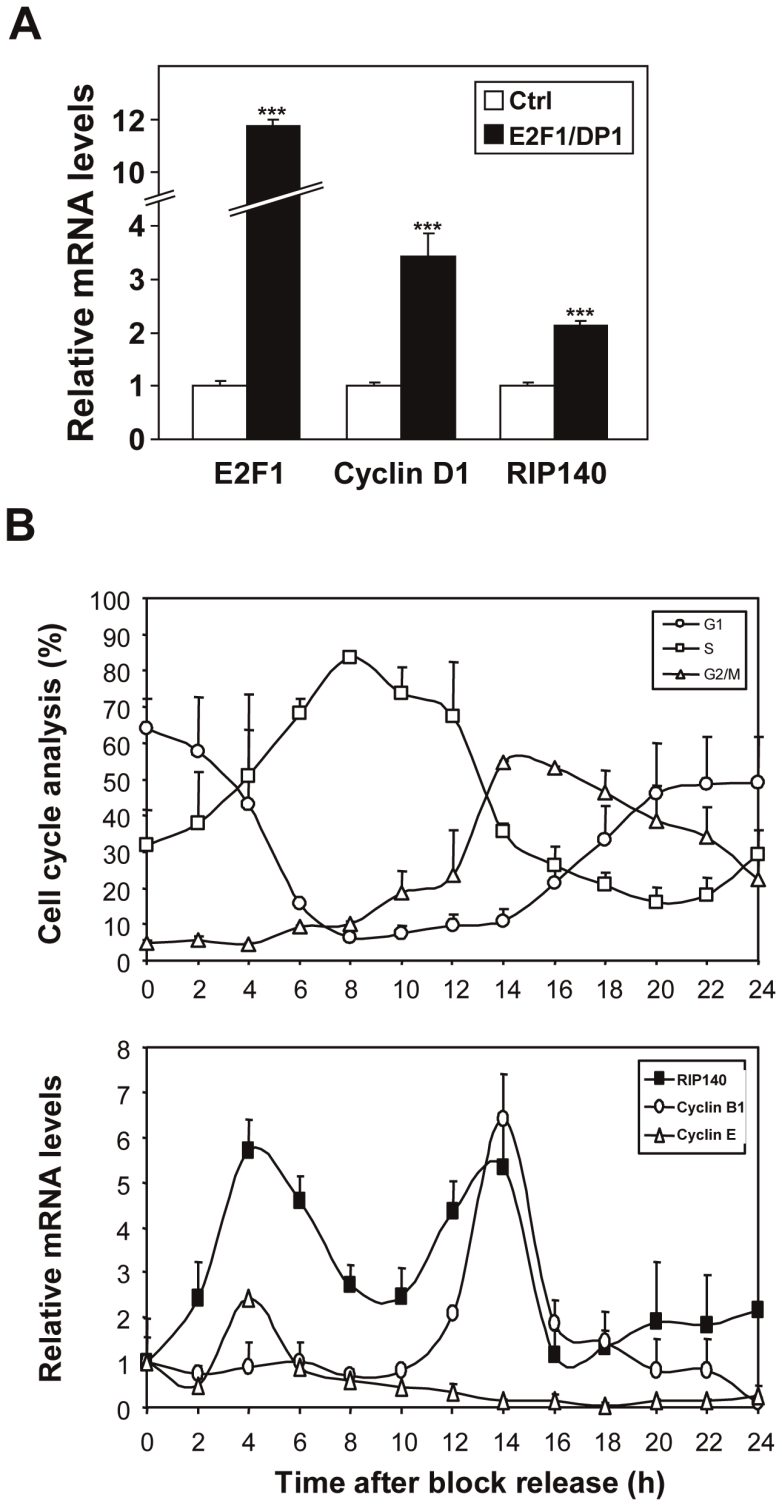
Regulation of the endogenous RIP140 gene. (**A**) The levels of E2F1, cyclin D1 and RIP140 mRNAs were measured in MCF-7 cells 2 days after transient transfection with plasmids allowing the overexpression of the E2F1/DP1 heterodimer. Statistical analysis was performed using the Student t test (***p<0.0001). (**B**) HeLa cells were synchronized by 2 mM hydroxyurea (HU) and, after block release the distribution of cells in G_1_, S and G_2_/M was analyzed by flow cytometry (upper panel). Cyclin B1 and E, and RIP140 mRNA levels were quantified by real-time quantitative RT-PCR as described in *Materials and Methods* (lower panel). The results are expressed in arbitrary units after normalization by RS9 mRNA levels. Values are the means ± S.D. of three independent experiments.

To strengthen our results on the transiently transfected RIP140 promoter, we analyzed the regulation of the endogenous RIP140 mRNA upon overexpression of E2F1 and DP1 by transient transfection. As shown in Figure 3A, this led to a significant increase in the levels of RIP140 mRNA comparable to that of cyclin D1 measured in parallel, thus confirming that RIP140 is a transcriptional target of E2F1.

As a consequence of the transcriptional control by E2Fs, the RIP140 gene expression might be regulated during cell cycle progression. To test this hypothesis, we performed cell synchronization using hydroxyurea and measured, by quantitative RT-PCR, the expression of RIP140 mRNA after release of the block (Figure 3B). Cell synchronization was monitored by FACS analysis (data not shown) and quantification of cyclin E and cyclin B1 mRNA levels peaking respectively at the transitions between G_1_ and S and between S and G_2_/M (Figure 3B). Very interestingly, our data indicated that, in such experiments, the accumulation of RIP140 mRNA varied more than 5-fold, with two peaks of accumulation at 4 and 14 hr after the block release which matched perfectly with the transient increases of cyclin E and B1 mRNAs respectively. These results therefore identified RIP140 as a novel cell-cycle regulated gene in human cancer cells.

### Localization of the Regulatory Elements

**Figure 4:**
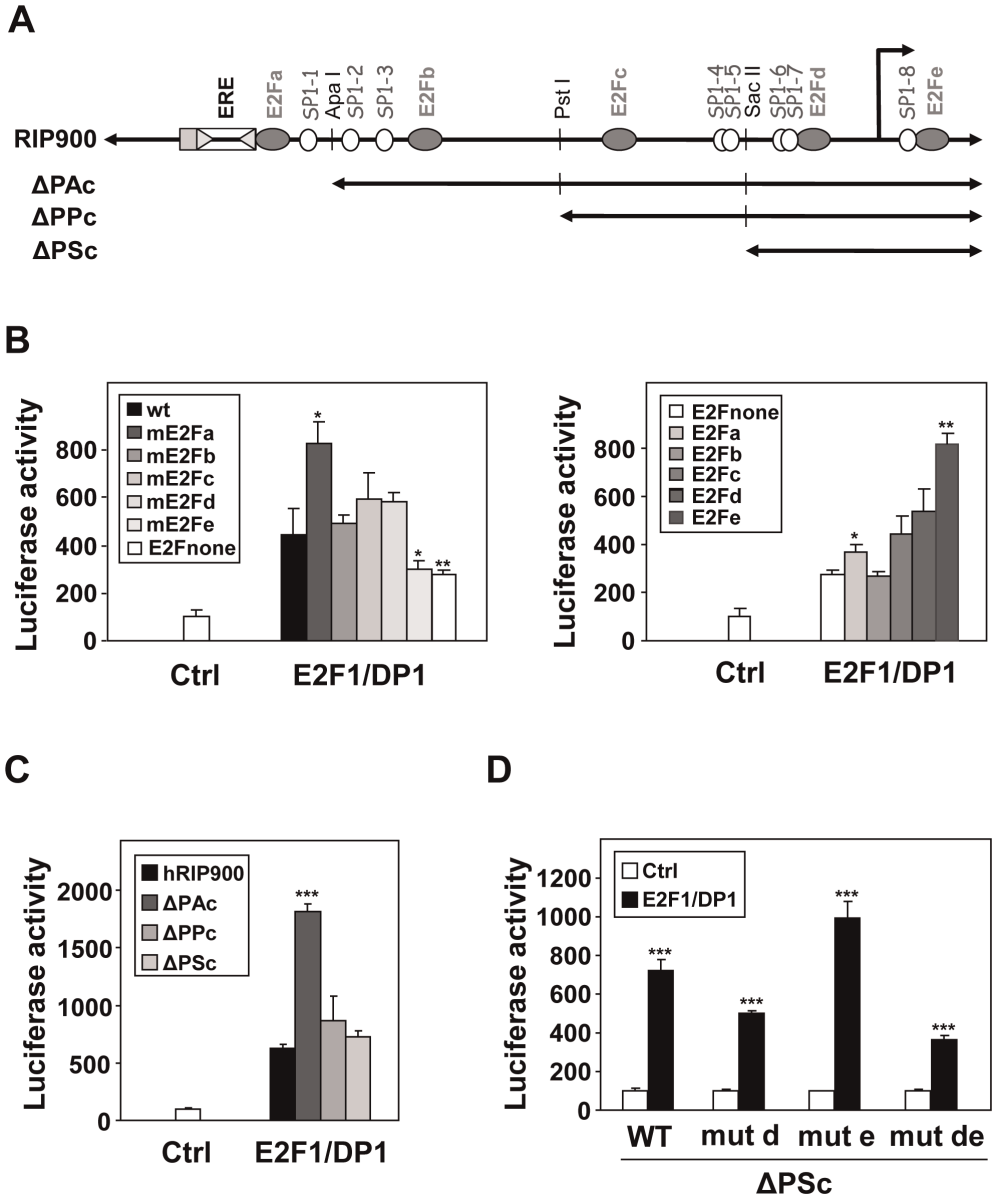
Importance of the proximal region of the RIP140 promoter. (**A**) Schematic representation of the RIP140 promoter sequence (RIP900 plasmid) showing the E2Fs binding sites (a, b, c, d and e, indicated with grey ovals), the Sp1 binding sites (open circles) and the different 5′ deletion mutants (ΔPAc, ΔPPc and ΔPSc). (**B**) MCF-7 cells were transiently transfected with the human RIP140 promoter reporter plasmids (25 ng) containing mutations of the E2Fs binding sites together with expression vectors for E2F1 and DP1 (25 ng each). In the left panel, each mutant has one E2F site mutated (mE2Fa, b, c, d and e) or multiple mutations which abolish all sites (E2Fnull). In the right panel, only the indicated site remains intact (E2Fa, b, c, d and e). (**C** and **D**) The same experiments as above were repeated with deletion mutants (respectively ΔPAc, ΔPPc, ΔPSc) of the RIP140 promoter (**C**) or with the proximal promoter region (mutant ΔPSc) with point mutations of the E2F binding sites (mE2Fd and e) (**D**). All results were expressed as described in Figure 2D and representative experiments are shown (n = 3).

Using promoter mutagenesis (deletion and point mutations), we then defined the cis-acting elements required for the positive regulation by E2Fs. In a first step, we generated mutants of the RIP140 promoter with point mutations in the different E2F response elements (Figure 4A). When we analyzed the effect of each individual mutation (Figure 4B, left panel), we found that mutation of the E2Fa site slightly increased the response whereas mutation of the E2Fe site decreased the response to E2F1/DP1 overexpression. Surprisingly, a construct with mutation of the five E2F response elements (E2Fnull) was still significantly regulated by E2F1/DP1 to a level comparable to that obtained with the mE2Fe reporter.

We then analyzed reporter transactivation when a single functional E2F response element was left intact (Figure 4B, right panel). When compared to the promoter mutated on the five E2F response elements, we found that the E2Fb site was not functional whereas all the other sites produced a significant transactivation, the highest effect being obtained with the E2Fe site. Interestingly, this construct even supported a significantly higher transactivation by E2F1/DP1 than the wild-type reporter (compare left and right panels). Altogether, these data suggested 1) that other cis-acting elements than these five E2F binding sites mediate the regulation of the RIP140 promoter by E2Fs and 2) that the distal (E2Fa) and proximal (E2Fe) response element support opposite regulation by E2Fs (negative and positive effects, respectively).

Deletion analysis of the promoter confirmed these data (Figure 4C, Figure S1A and B). Indeed, the regulation by E2F1/DP1 was stronger with the ΔPAc construct which lacks only the E2Fa site, thus confirming a negative effect of this E2F response element. Moreover, the ΔPSc reporter construct which encompasses only the E2Fd and E2Fe sites exhibited the same regulation than the wild-type RIP900 construct confirming that the proximal region of the promoter was sufficient to mediate the regulation by E2Fs. However, although mutations of the two sites (E2Fd and e) in the ΔPSc reporter construct reduced the regulation of luciferase activity in response to E2F1/DP1 (Figure 4D), the ΔPSc reporter construct with the double mutation was still transactivated by E2F1/DP1 again suggesting that other mechanisms are involved.

### Role of DP1 and Sp1 Transcription Factors in the Regulation by E2Fs

**Figure 5:**
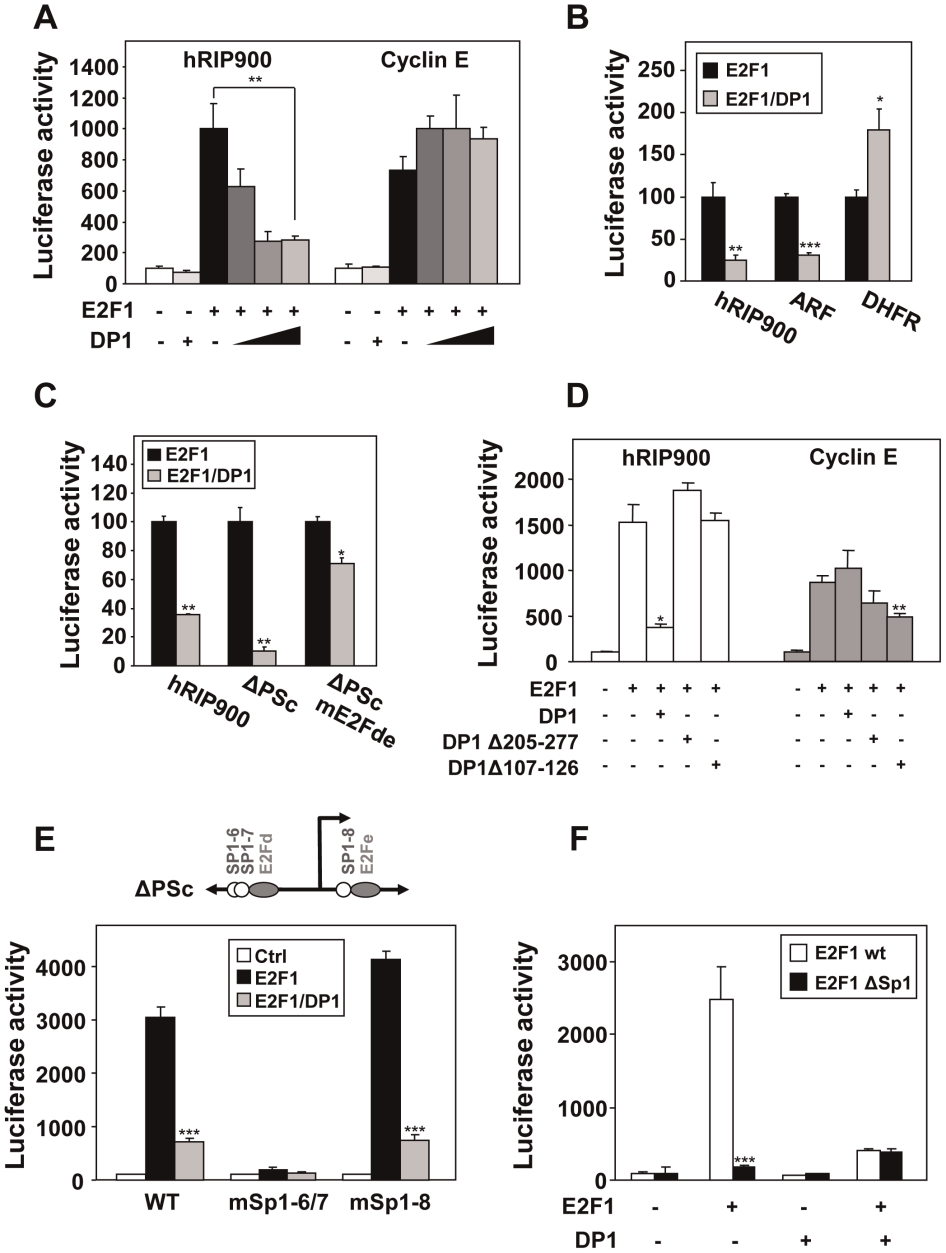
Effect of DP1 and Sp1 on the transactivation by E2F1. (**A**) MCF-7 cells were cotransfected respectively with the human RIP140 and cyclin E promoter reporter plasmids (25 ng) together with E2F1 (25 ng) and increasing dose of DP1 factors (0, 5, 25, 50 ng) (left). Results were expressed as in Figure 2D (n = 4). (**B**) The human RIP140, ARF and DHFR promoter reporter plasmids were tested with E2F1+/− DP1. The luciferase activity with E2F1 alone overexpressed was normalized at 100% (n = 3). (**C**) Different mutants of the RIP140 promoter (hRIP900wt, ΔPScwt, mE2Fde) were tested for the response to overexpression of E2F1+/− DP1. Results were expressed as in Figure 5B (n = 4). (**D**) The effect of two deletion mutants of DP1 protein (DP1 Δ107–126 that abolishes DNA binding and Δ205–277 for E2F binding) on E2F1 activity was measured on hRIP140 and cyclin E promoters. Results were expressed as in figure 2D (n = 3). (**E**) MCF-7 cells were transiently transfected as indicated in *Materials and Methods*, with the point mutants for Sp1 binding sites (#6/7 or #8 shown in Figure 3A) of the proximal sequence ΔPSc reporter plasmid (25 ng) together with expression vectors for E2F1+/− DP1 (25 ng each). Results were expressed as in Figure 2D (n = 3). **(F)** E2F1 mutated for Sp1 interaction (E2F1ΔSp1) was used +/− DP1 on the proximal promoter region (ΔPSc reporter plasmid), in the same conditions as above. Results were expressed as in Figure 2D (n = 3).

E2F transcription factors are believed to act as heterodimers with DP proteins which are ubiquitously expressed [22]. When we overexpressed only E2F1 (Figure 5A - black boxes), we observed a significant level of transactivation of the RIP140 promoter which might reflect the action of E2F1 as heterodimers with endogenous DP proteins. Similar transactivation upon overexpression of E2F1 only was also obtained for the cyclin E promoter. However, we found that coexpression of DP1 did not produce the same effect on the transactivation by E2F1 on the two promoters. Indeed, whereas we noticed an increase in the transactivation of the cyclin E reporter, DP1 expression vector cotransfection produced a strong inhibitory effect on the regulation of the RIP140 promoter by E2F1 (Figure 5A and Figure S1C).

To extend this observation, we performed similar transfection experiments on other E2F-target promoters *i.e.* the DHFR and ARF promoters. Interestingly, the negative effect observed with DP1 overexpression on the RIP140 promoter was also observed with the ARF promoter but not with the DHFR promoter (Figure 5B). As shown in Figure 5C, this effect of DP1 was also detected with the ΔPSc reporter construct and required the presence of the E2F binding sites since the inhibition by DP1 was strongly decreased with the ΔPSc reporter harboring a mutation of the E2Fd and e sites.

We then used different mutants of DP1 to further decipher the mechanisms involved in this effect. When DP1 is impaired in its ability to bind DNA (Δ107–126 mutant) [20] or to heterodimerize with E2F1 (Δ205–277 mutant) [21], we no longer observed the inhibitory effect of the transactivation by E2F1 on the RIP140 promoter (Figure 5D). By contrast, the two DP1 mutants exhibited a slight inhibitory effect on the cyclin E promoter when compared to the wild-type DP1. These data suggested that forcing heterodimerization of E2F1 with DP1 reduced the transcriptional response of the RIP140 promoter.

**Figure 6:**
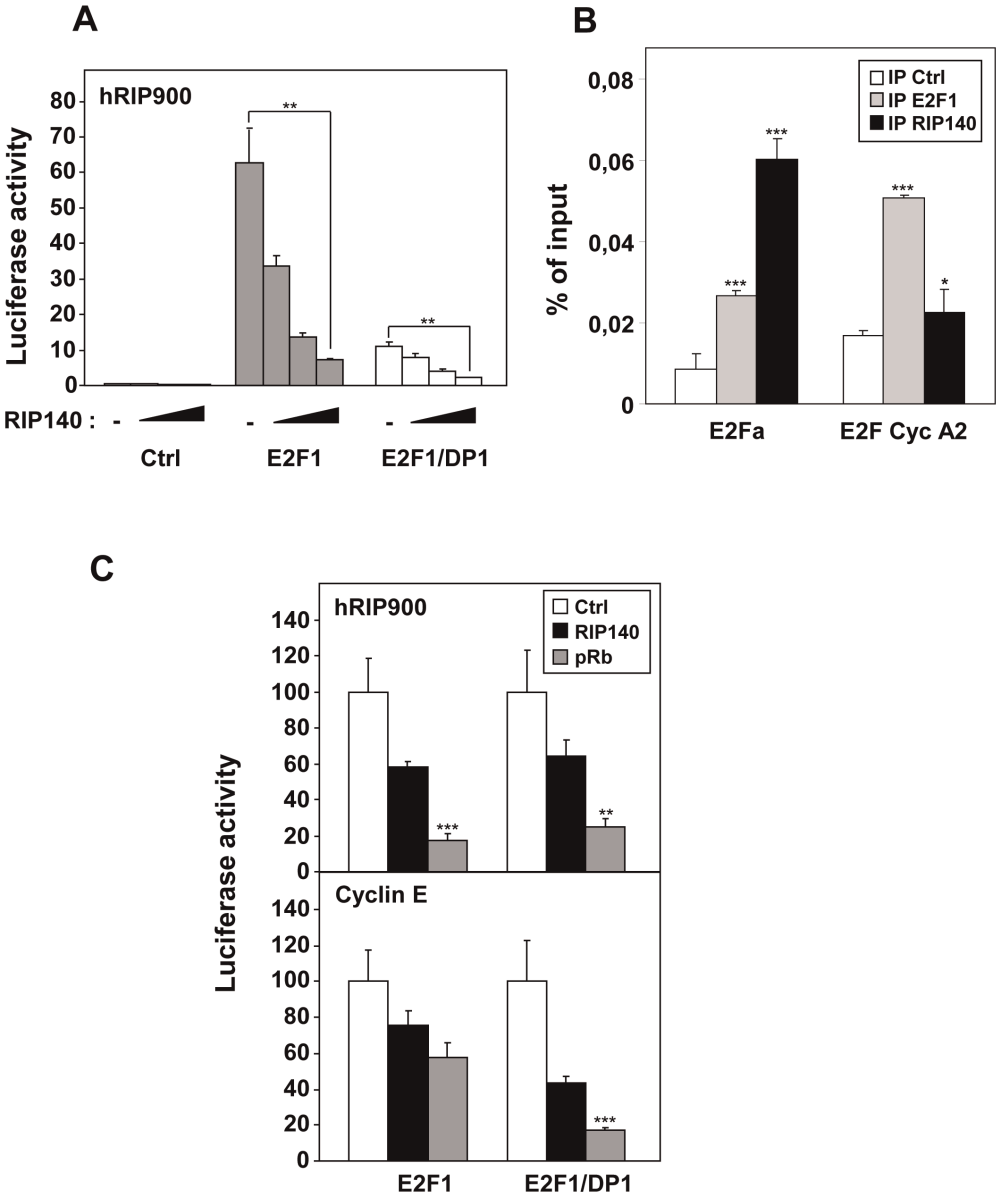
Repression of E2F transactivation by RIP140 and pocket proteins. (**A**) The human WT RIP900-luc reporter plasmid (25 ng) and E2F1+/− DP1 (25 ng each) were transiently transfected in MCF-7 cells with a dose response of RIP140 expression plasmids (0, 50, 100 or 200 ng). (**B**) ChIP experiments using immunoprecipitation (IP) of E2F1 and RIP140 on the E2Fa site of the human RIP140 promoter. The cyclin A2 promoter was used as a positive control. Data are expressed as percent of the PCR signal obtained with the amount of chromatin used for IP (input). Statistical analysis performed using the Student t test (***p<0.0001 and *p<0.05). (**C**) MCF-7 cells were transfected with human RIP900-luc or cyclin E-luc reporter plasmid (25 ng), with E2F1+/− DP1 (25 ng) and pocket proteins Rb or RIP140 expression vectors (200 ng or 250 ng) in 96 well plates in the indicated combinations. Results were expressed as in Figure 2D (n = 4).

Altogether, the results from promoter mutagenesis combined to the atypical effect of DP1 overexpression strongly supported a complex regulation of the RIP140 promoter by E2F1 involving for instance a combination of direct and indirect recruitment. One hypothesis for an indirect recruitment of E2F1 on the RIP140 involved Sp1 transcription factors. Indeed, several studies have reported a physical interaction and a transcriptional synergism between E2F1 and Sp1 [23]. The proximal region of the RIP140 promoter encompasses several functional Sp1 binding sites (see Figures 1B and 5E and [6]). We therefore introduced point mutations in the ΔPSc reporter construct which targeted the different Sp1 sites. As shown in Figure 5E, disruption of sites 6/7 totally abolished the regulation by E2F1 (with or without coexpression of DP1) whereas mutation of the Sp1 site 8 was ineffective. To further emphasize the role of Sp1 in the regulation of the RIP140 promoter by E2F1, we generated a mutant of E2F1 lacking the region from residue 102 to 125 which mediates its interaction with Sp1 [18]. When compared to wild-type E2F1, this mutant transactivated to the same extend when DP1 was cotransfected (*i.e.* when heterodimerization was forced) but was significantly less efficient to activate transcription from the RIP140 promoter when overexpressed alone *i.e.* when Sp1-mediated transactivation took place (Figure 5F). Same results were obtained on the proximal promoter region of the RIP140 gene using the ΔPSc reporter construct (data not shown). Altogether, this indicated that the positive regulation of the RIP140 promoter by E2F1 involved the proximal region spanning from nucleotides −140 to +100. This regulation implicated a classical recruitment of E2F1 (mainly through the E2Fe site) and an indirect recruitment or a stabilization of this binding *via* Sp1.

### Existence of a Negative Regulatory Loop Involving RIP140 and E2F1

We and others (see [1] for a review) previously reported that RIP140 was engaged in several negative feedback regulatory loops involving various nuclear receptors. We also recently described the inhibitory role of RIP140 in the regulation of E2F1 activity [9]. Since our above-mentioned results demonstrated that E2F1 was a potent regulator of RIP140 expression, we investigated whether RIP140 could in turn control its own activation by E2F1. As shown in Figure 6A, RIP140 overexpression led to an inhibition of the transactivation of its own promoter by E2F1 either when overexpressed alone or in combination with DP1. Using chromatin immunoprecipitation, we confirmed the presence of RIP140 on the region of its own promoter which encompasses the distal E2Fa binding site. As shown in Figure 6B, the signal obtained after immunoprecipitation of RIP140 was even stronger than that obtained on the cyclin A2 promoter. In parallel, we compared the regulation of E2F1 transactivation by RIP140 or pRb both on the RIP140 and cyclin E promoters. As shown in Figure 6C, we found that pRb was significantly more potent than RIP140 to inhibit E2F1 activity on both reporters, except on the cyclin E promoter in the presence of E2F1 alone.

### Effect of E2F1 Knock-out on RIP140 Expression during Adipocyte Differentiation

In addition to the control of cell proliferation and apoptosis, E2F1 has recently been shown to play critical role in metabolic control and in particular to positively regulates adipogenesis [14]. For instance, it has been reported that lipid incorporation is decreased in E2F1 knock-out MEFs stimulated to differentiate into adipocytes [24]. RIP140 is also a key regulator of fat metabolism [25] and, interestingly, both E2F1 [24] and RIP140 [26] expression increased during the differentiation process, E2F1 being induced earlier than RIP140. In order to determine whether E2F1 participates in the regulation of RIP140 expression during adipocyte differentiation, we stimulated both E2F1 wild-type and knock-out MEFs to differentiate *in vitro* in response to hormone stimulation.

**Figure 7:**
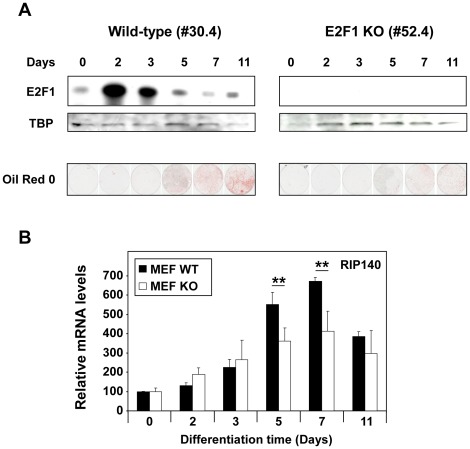
Regulation of RIP140 mRNA levels in E2F1 knock-out mice. (**A**) Adipocyte differentiation of mouse embryo fibroblasts. At different times of adipocyte differentiation, lipid accumulation was measured by Oil Red O staining in wild-type (WT) and E2F1^−/−^ MEFs. The levels of E2F1 protein were detected by western-blot analysis as described in *Materials and Methods* and loading control was performed using an anti-TBP antibody. Time 0 corresponds to the addition of the differentiation medium. (**B**) Analysis of RIP140 mRNA expression by RT-qPCR in wild-type (WT) and E2F1^−/−^ MEFs. Quantification were performed at the same times of adipocyte differentiation as in panel A. Data are expressed as percent of the values obtained at day 0. Statistical analysis was performed using the Student t test (**p<0.005).

As shown in Figure 7A and as expected, E2F1 expression transiently increased during differentiation (peak after 2 days) and is totally lost in E2F1 knock-out mice. In addition, differentiation followed by Oil Red O staining to detect lipid droplets was significantly reduced in E2F1 knock-out MEFs as compared to E2F1 wild-type cells. We quantified RIP140 mRNA levels in parallel and observed a peak of expression at day 7 post-differentiation as previously described [26]. Importantly, RIP140 mRNA accumulation was significantly reduced in knock-out cells supporting a role of E2F1 in the control of RIP140 expression (Figure 7B).

## Discussion

RIP140 was initially characterized as a transcriptional coregulator of ligand-activated nuclear hormone receptors, involved in the control of ovarian functions and metabolic pathways (for a review, see [27]). In the present study, we identified RIP140 as a novel cell-cycle regulated gene whose expression is directly controlled by E2F transcription factors.

Based on *in vitro* DNA-protein interaction assays, ChIP experiments and transient transfection assays, our data clearly demonstrate that E2F1 (as well as other activating E2Fs) increase transcription from the RIP140 promoter through binding to the proximal promoter region. Several evidences (mutagenesis of Sp1 response elements and use of an E2F1 mutant defective for Sp1 binding) strongly supported the involvement of Sp1 transcription factors in the regulation of RIP140 by E2F1. In addition, the use of mithramycin which has been described as an inhibitor of Sp1 binding to DNA [28] also inhibited the regulation of the RIP140 promoter by E2F1 (data not shown).

The interaction between Sp1 and E2F1 has been previously demonstrated *in vitro* and by coimmunoprecipitation [29]–[30] and amino acids 102–125 of E2F-1 and 622–668 of Sp1 appeared sufficient for interaction of the two proteins [18]. Interestingly, it has been reported that the expression of the SRC3 gene (which encodes a transcriptional coregulator of both nuclear receptors [31] and E2F1 [32]) was also controlled by E2F1 acting *via* Sp1 sites [33]. Interestingly, using ChIP-chip assays and high density oligonucleotide tiling arrays, Bieda *et al*. have shown that the great majority of E2F1 binding sites are in CpG islands (which are highly enriched in Sp1 sites) and lack the consensus binding site motif [34]. It is therefore possible that on the RIP140 promoter, Sp1 not only synergizes with E2F1 but directly participates in the recruitment of E2F1 by protein-protein interaction. The overexpression of E2F1 impaired in its ability to bind DNA (E132 mutant) [19] resulted in a dramatic decrease in transactivation of the RIP140 promoter in the absence or presence of overexpressed DP1 (Figure S2) suggesting that the DNA-binding domain of E2F1 could be required for both direct and indirect recruitment on the RIP140 promoter. This is in agreement with the fact that the DNA-binding region of E2F1 overlaps in part with the domain which binds to Sp1 [18].

An indirect recruitment of E2F1 is supported by our observation highlighting the repressive role of DP1 overexpression in the regulation of RIP140 promoter. This effect is also observed with the mouse RIP140 promoter (Figure S3A) and appears restricted to certain promoters since it was also observed on the ARF promoter construct but not with other promoters such as cyclin E or DHFR, thus emphasizing the specificity of this regulation. This observation is reminiscent of previous work showing that upon DP1 knock-down in cells, the expression of some E2F-target genes such as PCNA and MCM3 is not inhibited [35]. The use of DP1 mutants indicates that both the DNA-binding domain and the heterodimerization interface were required to achieve this regulation. The reduced transcriptional response of the RIP140 promoter to E2F1 when DP1 is overexpressed may thus result from a forcing of E2F1 heterodimerization and/or direct competition of DP1 with Sp1 for the interaction with E2Fs, both leading to a decreased synergism between the latter partners.

Altogether, our *in vitro* results clearly demonstrate that RIP140 is a direct target of E2F1. This conclusion is reinforced by our data demonstrating that the accumulation of RIP140 mRNA varies during cell cycle progression although it remains to be demonstrated whether E2Fs are involved in the two peaks of RIP140 expression observed in synchronized cells at the G1/S and G2/M transition. This is conceivable since recent data from Nevins’ laboratory clearly demonstrated that E2Fs are key actors in gene regulation during the G2/M transition [36]. Indeed, the analysis of the G2-regulated cdc2 and cyclin B1 genes revealed the presence of both positive- and negative acting E2F response elements which interact with distinct E2Fs (activators or repressors). A similar implication of different E2F elements, binding to distinct E2Fs and relaying positive or negative regulation of transcription, has been reported for the E2F1 promoter [37]. Data presented in Figure 4B, 4C and S1C indicated that deletion or mutation of the distal site E2Fa in the RIP140 promoter leads to a stronger induction by E2F1/DP1. As shown in Figure S4, E2F4 is able to interact with the E2Fa site in gel shift assay (panel A) and in ChIP experiments (panel B), the interaction being even stronger than that obtained with E2F1. Moreover, the deletion of the distal region of the RIP140 promoter which encompassed the E2Fa site (ΔPAc reporter construct) significantly decreased the repression of E2F1 transactivation by E2F4 (Figure S4C). Altogether, this suggests that the distal E2F response element might play a role in the regulation of the RIP140 promoter by E2Fs by preferentially recruiting repressive E2Fs such as E2F4.

Interestingly, a bioinformatic analysis was conducted on the RIP140 promoter sequence using six different species (namely *Homo sapiens, Pan troglodytes, Bos Taurus, Oryctolagus cuniculus, Mus musculus and Rattus norvegicus*). As shown in Figure S5, data indicated that the response elements which are the best conserved during evolution, are those that we found to be the most relevant from a functional point of view. Indeed, the Sp1 sites #6–7 and the E2Fd site are perfectly well conserved in the six different species. By contrast, the other binding sites, namely E2Fb, E2Fc and E2Fe, are very poorly conserved. It should be noted that the E2Fa site is well conserved in 3 species and that an upstream site is detected at −877 in mouse and rat supporting the functionality of this distal site that we evidenced in the human promoter based on mutation analysis (see Figure 4B and C).

We and others previously reported that several nuclear receptors (such as ERα [6], AR [5] and RARα [38]) or other transcription factors (such as AhR [6]) positively regulate RIP140 mRNA levels, thus evidencing the existence of several negative feedback loops. Such a regulatory loop also exists with E2F1 whose transcriptional activity is negatively regulated by RIP140 on various promoters [9] and also on the RIP140 promoter itself (Figure 6). The RIP140 gene is therefore controlled both by ERα and E2Fs thus extending the list of coregulated genes (CDC6, CDC25A, PCNA, POLA2, RFC4, SMC2, PRC1) and confirming a previous observation made by the Mader’s laboratory which reported that one of the most enriched binding sites in up-regulated estrogen target genes is that for E2F transcription factors [39].

From a more physiological point of view, E2F1 has been described as a multifaceted transcription factor which can both promote and inhibit cell proliferation and tumorigenesis [12]. More recently, its implication in different metabolic processes including lipid and adipocyte metabolism or glucose homeostasis has been reported [14]. Our data suggest that RIP140 might play a role as an E2F1 target gene in the control of adipocyte differentiation (Figure 7). Further work will be necessary to precisely define the role of RIP140 in E2F biological activities. This will require in particular the phenotypic analysis of transgenic mice with combined altered expression of E2F1 and RIP140.

In conclusion, this work is the first report to provide *in vitro* and *in vivo* evidences demonstrating that the E2F pathway exerts at direct transcriptional control on RIP140 expression and that this regulation may play an important role in physiological responses to E2F1 on key processes such as proliferation, apoptosis or differentiation which are strongly disturbed in cancer or metabolic diseases.

## Supporting Information

Figure S1 **Regulation of the human RIP140 promoter by E2F1.** (A and B) MCF-7 cells were transiently transfected with the different human RIP140 promoter reporter plasmids (RIP900, ΔPAc or ΔPSc) together or not (Ctrl) with expression vectors for E2F1 and DP1. Relative luciferase activities are expressed as percent of control and are the mean (±SD) of several independent experiments (n = 5). (C) MCF-7 cells were transiently transfected with the human RIP900 reporter plasmids together with expression vectors for E2F1 in the presence or absence of DP1. Relative luciferase activities are expressed as percent of control and are the mean (±SD) of several independent experiments (n = 10). The paired t-test was used for statistical analysis.
(TIF)

Figure S2 **Effect of the E2F1 mutant (E132) on the human RIP140 and cyclin E promoters.** (A) MCF-7 cells were transiently transfected with the human RIP140 or cyclin E promoter reporter plasmids (25 ng) together with expression vectors for E2F1wt or E132 mutant and DP1 (25 ng each). Relative luciferase activity was normalized with renilla luciferase activity as described in Materials and Methods. The values are expressed as percent of control and are the mean (±SD) of triplicate. The Student t-test was used for statistical analysis. (B) The expression of the different plasmids used in panel A was controlled by Western-blot as described in Material and Methods. All the tracks shown are from the same western-blot but the third track (E132) has been cut and paste to generate the Figure.
(TIF)

Figure S3 **Effect of activator E2Fs and DP1 on the murine RIP140 promoter.** (A) MCF-7 cells were transiently transfected with the murine RIP140 promoter reporter plasmids (25 ng) together with expression vectors for E2F1, E2F2, E2F3 and DP1 (25 ng each). Results are expressed as described in legend of Figure S2A (n = 3). The values are expressed as percent of control. (B) (C) The expression of DP1 and that of the three E2F plasmids used in panel A was controlled by Western-blot as described in Material and Methods.
(TIF)

Figure S4 **Binding of E2F4 on the distal site of the RIP140 promoter.** (A) Electromobility shift assay was used to analyze E2F1/DP1 or E2F4/DP1 binding on the adenoviral E2F response element (Ad2E2F) or on the E2Fa site of the RIP140 promoter (see Figure 1). (B) ChIP experiments using immunoprecipitation (IP) of E21and E2F4 on the E2Fa site of the human RIP140 promoter. Data are expressed as percent of the PCR signal obtained with the amount of chromatin used for IP (input). Negative control of IP was done using an isotype-matched mAb. (C) MCF-7 cells were transiently transfected with the human and mutant ΔPAc RIP140 promoter reporter plasmids (25 ng) together with expression vectors for E2F1 and DP1 (25 ng each) and increasing amounts of E2F4 (0/5/50 ng). Relative luciferase activity was normalized with renilla luciferase activity as described in Materials and Methods, and is the mean (±SD) of triplicate. The values are expressed as a percentage of the activity obtained with control.
(TIF)

Figure S5 **Conservation of the NRIP1 promoter sequence among mammals.** The NRIP1 promoter regions of six mammalian species (from exon 1 b up to about 1 kbp) have been aligned using the Multalin program (hosted at http://multalin.toulouse.inra.fr/multalin/). Genome assembly used for each species were Homo sapiens (hg19), Pan troglodytes (panTro3), Bos taurus (bosTau6), Oryctolagus cuniculus (oryCun2), Mus musculus (mm9), and Ratus norvegicus (rn4) as indicated at the left of the aligned sequences that were extracted from the UCSC genome browser (http://genome.ucsc.edu/). Localization of putative E2F and Sp1 transcription binding sites was performed using MathInspector pattern search program from Genomatix (http://www.genomatix.de). E2F and Sp1 binding sites are shown in grey and white boxes respectively, with names above the sequence according to Figure 1 b and prefixed with h or m to distinguish human and mouse sites. The ERE that we [6] and others [7] found in both human and mouse promoters is shown as a landmark. Coordinates shown above the sequence are global to the alignment and are relative to the beginning of exon 1 b (large box at the end of the alignment).
(TIF)

Table S1 **Oligonucleotide sequences.** The table shows the sequences of all the oligonucleotides used in the different assays (ChIP, gel shift assays and Q-PCR). The corresponding species (human or mouse) is indicated as well as the orientation of oligonucleotides *i.e.* sense/forward (fwd) or reverse (rev). The name of the target (promoter, binding site or mRNA) is also presented.
(TIF)

## References

[1] Augereau P, Badia E, Carascossa S, Castet A, Fritsch S (2006). The nuclear receptor transcriptional coregulator RIP140.. Nucl Recept Signal.

[2] Huq MM, Wei LN (2005). Post-translational modification of nuclear co-repressor receptor interacting protein 140 by acetylation.. Mol Cell Proteomics.

[3] Yang XJ, Seto E (2008). Lysine acetylation: codified crosstalk with other posttranslational modifications.. Mol Cell.

[4] Thenot S, Charpin M, Bonnet S, Cavailles V (1999). Estrogen receptor cofactors expression in breast and endometrial human cancer cells.. Mol Cell Endocrinol.

[5] Carascossa S, Gobinet J, Georget V, Lucas A, Badia E (2006). Receptor-interacting protein 140 is a repressor of the androgen receptor activity.. Mol Endocrinol.

[6] Augereau P, Badia E, Fuentes M, Rabenoelina F, Corniou M (2006). Transcriptional regulation of the human NRIP1/RIP140 gene by estrogen is modulated by dioxin signalling.. Mol Pharmacol.

[7] Nichol D, Christian M, Steel JH, White R, Parker MG (2006). RIP140 expression is stimulated by ERRalpha during adipogenesis.. J Biol Chem.

[8] Steel JH, White R, Parker MG (2005). Role of the RIP140 corepressor in ovulation and adipose biology.. J Endocrinol.

[9] Docquier A, Harmand PO, Fritsch S, Chanrion M, Darbon JM (2010). The transcriptional coregulator RIP140 represses E2F1 activity and discriminates breast cancer subtypes.. Clin Cancer Res.

[10] Iaquinta PJ, Lees JA (2007). Life and death decisions by the E2F transcription factors.. Curr Opin Cell Biol.

[11] Tsantoulis PK, Gorgoulis VG (2005). Involvement of E2F transcription factor family in cancer.. Eur J Cancer.

[12] Chen HZ, Tsai SY, Leone G (2009). Emerging roles of E2Fs in cancer: an exit from cell cycle control.. Nat Rev Cancer.

[13] Wu Z, Zheng S, Yu Q (2009). The E2F family and the role of E2F1 in apoptosis.. Int J Biochem Cell Biol.

[14] Blanchet E, Annicotte JS, Fajas L (2009). Cell cycle regulators in the control of metabolism.. Cell Cycle.

[15] DeGregori J, Johnson DG (2006). Distinct and Overlapping Roles for E2F Family Members in Transcription, Proliferation and Apoptosis.. Curr Mol Med.

[16] Blais A, Dynlacht BD (2007). E2F-associated chromatin modifiers and cell cycle control.. Curr Opin Cell Biol.

[17] Polager S, Ginsberg D (2008). E2F – at the crossroads of life and death.. Trends Cell Biol.

[18] Rotheneder H, Geymayer S, Haidweger E (1999). Transcription factors of the Sp1 family: interaction with E2F and regulation of the murine thymidine kinase promoter.. J Mol Biol.

[19] Hsieh JK, Fredersdorf S, Kouzarides T, Martin K, Lu X (1997). E2F1-induced apoptosis requires DNA binding but not transactivation and is inhibited by the retinoblastoma protein through direct interaction.. Genes Dev.

[20] Wu CL, Classon M, Dyson N, Harlow E (1996). Expression of dominant-negative mutant DP-1 blocks cell cycle progression in G1.. Mol Cell Biol.

[21] Datta A, Nag A, Raychaudhuri P (2002). Differential regulation of E2F1, DP1, and the E2F1/DP1 complex by ARF.. Mol Cell Biol.

[22] Hitchens MR, Robbins PD (2003). The role of the transcription factor DP in apoptosis.. Apoptosis.

[23] Wierstra I (2008). Sp1: emerging roles–beyond constitutive activation of TATA-less housekeeping genes.. Biochem Biophys Res Commun.

[24] Fajas L, Landsberg RL, Huss-Garcia Y, Sardet C, Lees JA (2002). E2Fs regulate adipocyte differentiation.. Dev Cell.

[25] White R, Morganstein D, Christian M, Seth A, Herzog B (2008). Role of RIP140 in metabolic tissues: connections to disease.. FEBS Lett.

[26] Leonardsson G, Steel JH, Christian M, Pocock V, Milligan S (2004). Nuclear receptor corepressor RIP140 regulates fat accumulation.. Proc Natl Acad Sci U S A.

[27] Christian M, White R, Parker MG (2006). Metabolic regulation by the nuclear receptor corepressor RIP140.. Trends Endocrinol Metab.

[28] Blume SW, Snyder RC, Ray R, Thomas S, Koller CA (1991). Mithramycin inhibits SP1 binding and selectively inhibits transcriptional activity of the dihydrofolate reductase gene in vitro and in vivo.. J Clin Invest.

[29] Karlseder J, Rotheneder H, Wintersberger E (1996). Interaction of Sp1 with the growth- and cell cycle-regulated transcription factor E2F.. Mol Cell Biol.

[30] Lin SY, Black AR, Kostic D, Pajovic S, Hoover CN (1996). Cell cycle-regulated association of E2F1 and Sp1 is related to their functional interaction.. Mol Cell Biol.

[31] Liao L, Kuang SQ, Yuan Y, Gonzalez SM, O’Malley BW (2002). Molecular structure and biological function of the cancer-amplified nuclear receptor coactivator SRC-3/AIB1.. J Steroid Biochem Mol Biol.

[32] Louie MC, Zou JX, Rabinovich A, Chen HW (2004). ACTR/AIB1 functions as an E2F1 coactivator to promote breast cancer cell proliferation and antiestrogen resistance.. Mol Cell Biol.

[33] Mussi P, Yu C, O’Malley BW, Xu J (2006). Stimulation of steroid receptor coactivator-3 (SRC-3) gene overexpression by a positive regulatory loop of E2F1 and SRC-3.. Mol Endocrinol.

[34] Bieda M, Xu X, Singer MA, Green R, Farnham PJ (2006). Unbiased location analysis of E2F1-binding sites suggests a widespread role for E2F1 in the human genome.. Genome Res.

[35] Maehara K, Yamakoshi K, Ohtani N, Kubo Y, Takahashi A (2005). Reduction of total E2F/DP activity induces senescence-like cell cycle arrest in cancer cells lacking functional pRB and p53.. J Cell Biol.

[36] Zhu W, Giangrande PH, Nevins JR (2004). E2Fs link the control of G1/S and G2/M transcription.. EMBO J.

[37] Araki K, Nakajima Y, Eto K, Ikeda MA (2003). Distinct recruitment of E2F family members to specific E2F-binding sites mediates activation and repression of the E2F1 promoter.. Oncogene.

[38] Kerley JS, Olsen SL, Freemantle SJ, Spinella MJ (2001). Transcriptional activation of the nuclear receptor corepressor RIP140 by retinoic acid: a potential negative-feedback regulatory mechanism.. Biochem Biophys Res Commun.

[39] Bourdeau V, Deschenes J, Laperriere D, Aid M, White JH (2008). Mechanisms of primary and secondary estrogen target gene regulation in breast cancer cells.. Nucleic Acids Res.

