# Supplementary figures and images for: The RIP140 Gene Is a Transcriptional Target of E2F1
Source: PLoS One. 2012 May 18;7(5):e35839. doi: 10.1371/journal.pone.0035839 (PMC3356364; doi:10.1371/journal.pone.0035839)

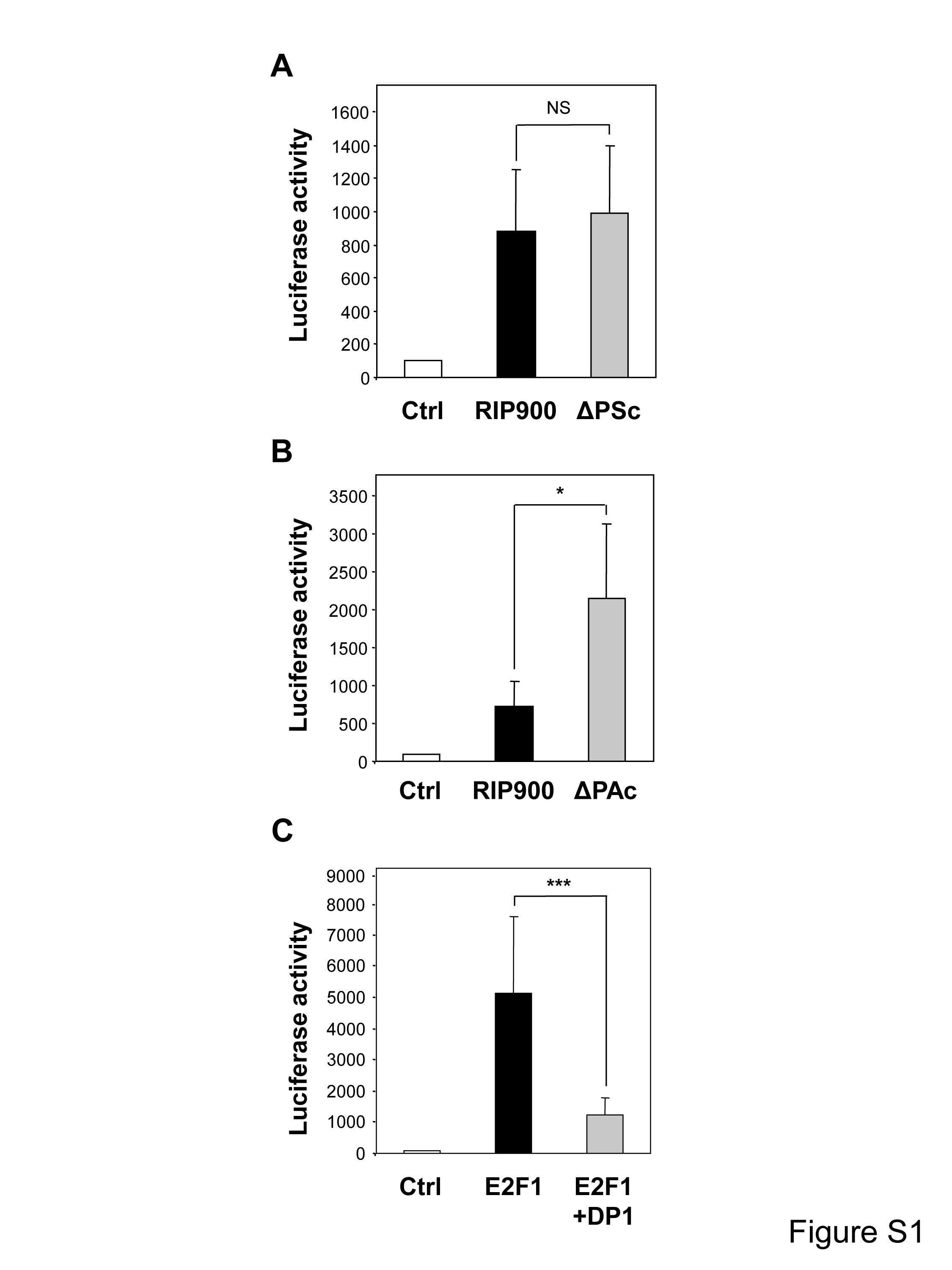

Supplement: Figure S1 — Regulation of the human RIP140 promoter by E2F1. (A and B) MCF-7 cells were transiently transfected with the different human RIP140 promoter reporter plasmids (RIP900, ΔPAc or ΔPSc) together or not (Ctrl) with expression vectors for E2F1 and DP1. Relative luciferase activities are expressed as percent of control and are the mean (±SD) of several independent experiments (n = 5). (C) MCF-7 cells were transiently transfected with the human RIP900 reporter plasmids together with expression vectors for E2F1 in the presence or absence of DP1. Relative luciferase activities are expressed as percent of control and are the mean (±SD) of several independent experiments (n = 10). The paired t-test was used for statistical analysis. (TIF) [file pone.0035839.s001.tif]

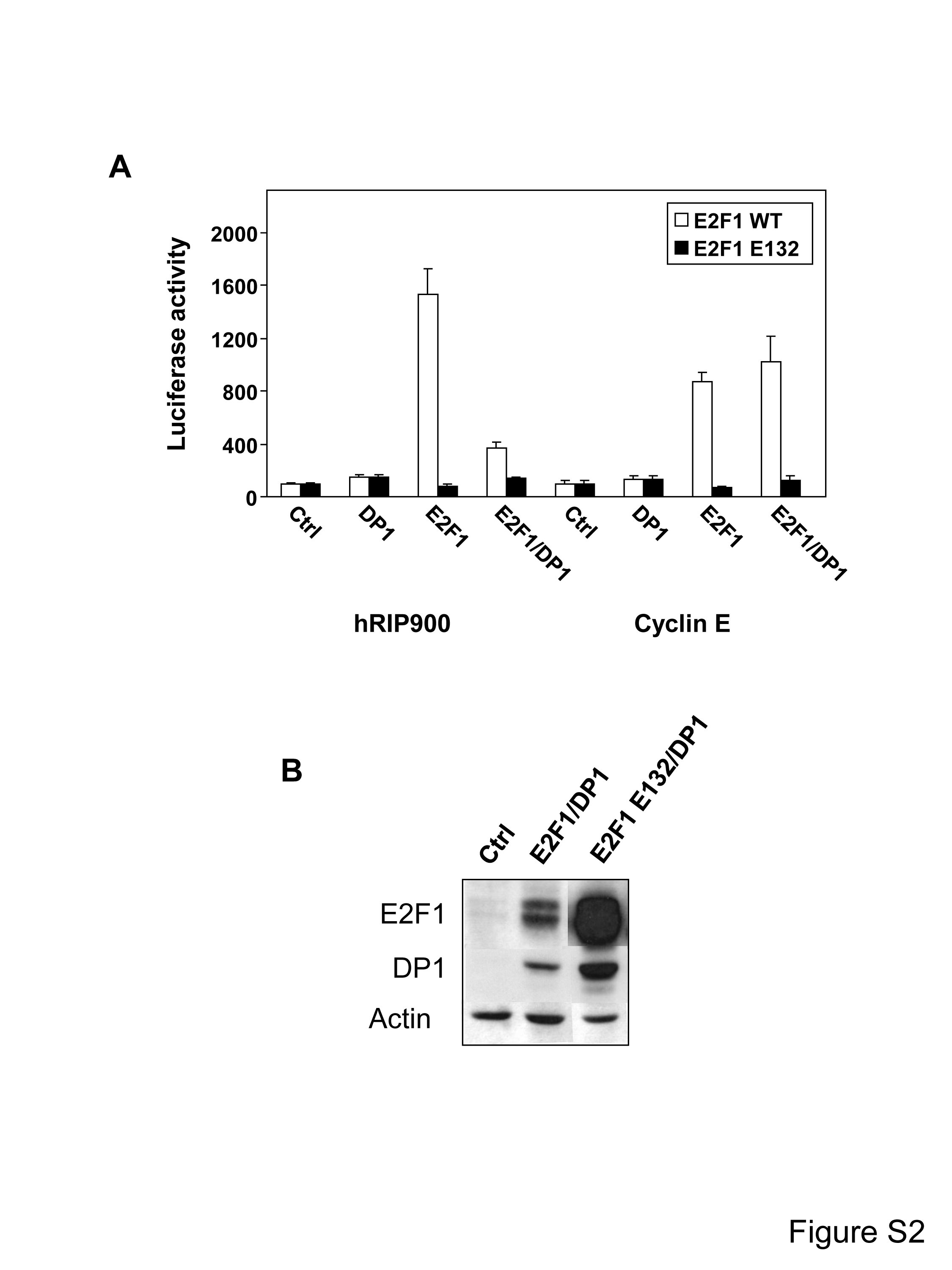

Supplement: Figure S2 — Effect of the E2F1 mutant (E132) on the human RIP140 and cyclin E promoters. (A) MCF-7 cells were transiently transfected with the human RIP140 or cyclin E promoter reporter plasmids (25 ng) together with expression vectors for E2F1wt or E132 mutant and DP1 (25 ng each). Relative luciferase activity was normalized with renilla luciferase activity as described in Materials and Methods. The values are expressed as percent of control and are the mean (±SD) of triplicate. The Student t-test was used for statistical analysis. (B) The expression of the different plasmids used in panel A was controlled by Western-blot as described in Material and Methods. All the tracks shown are from the same western-blot but the third track (E132) has been cut and paste to generate the Figure. (TIF) [file pone.0035839.s002.tif]

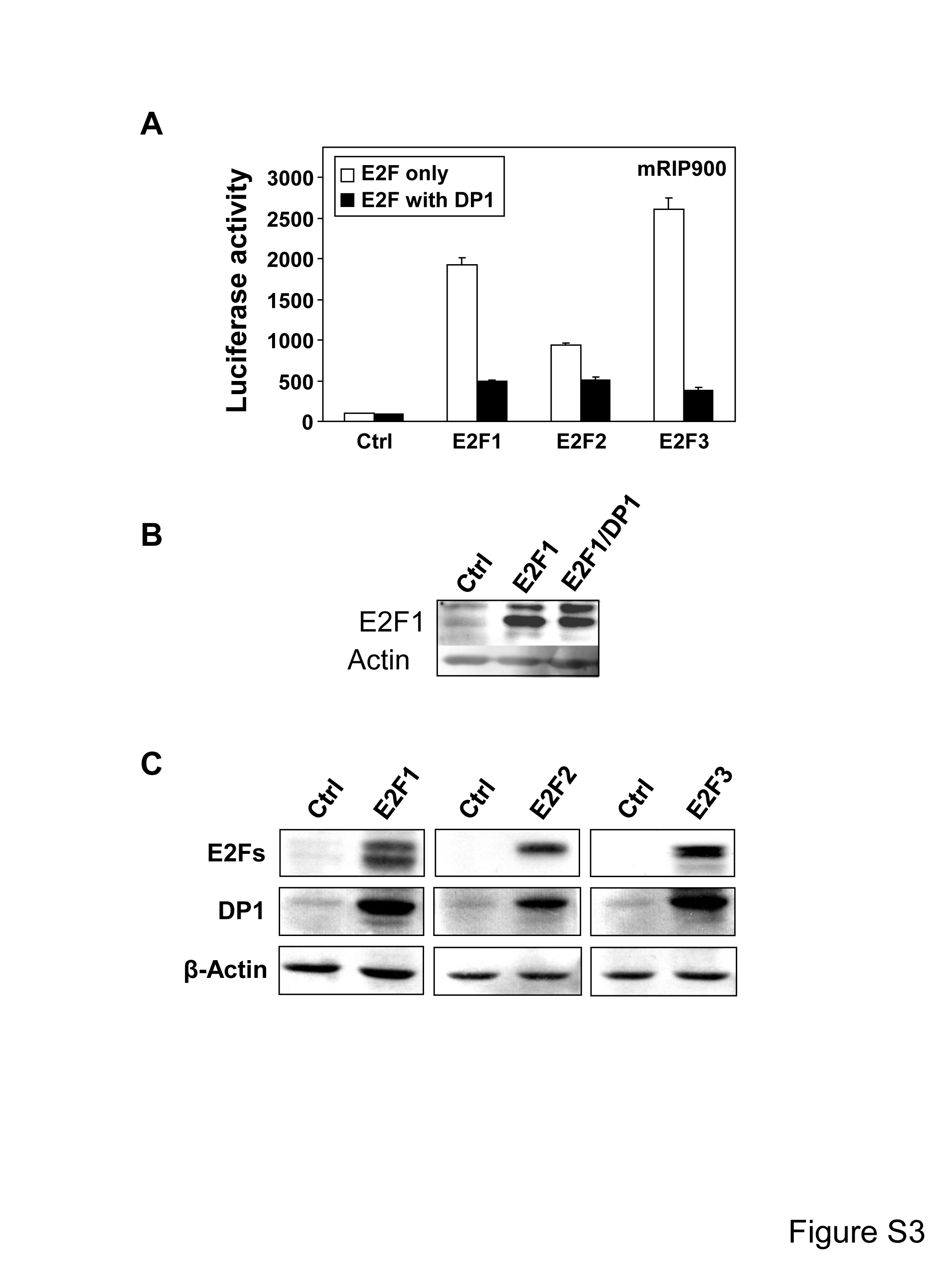

Supplement: Figure S3 — Effect of activator E2Fs and DP1 on the murine RIP140 promoter. (A) MCF-7 cells were transiently transfected with the murine RIP140 promoter reporter plasmids (25 ng) together with expression vectors for E2F1, E2F2, E2F3 and DP1 (25 ng each). Results are expressed as described in legend of Figure S2A (n = 3). The values are expressed as percent of control. (B) (C) The expression of DP1 and that of the three E2F plasmids used in panel A was controlled by Western-blot as described in Material and Methods. (TIF) [file pone.0035839.s003.tif]

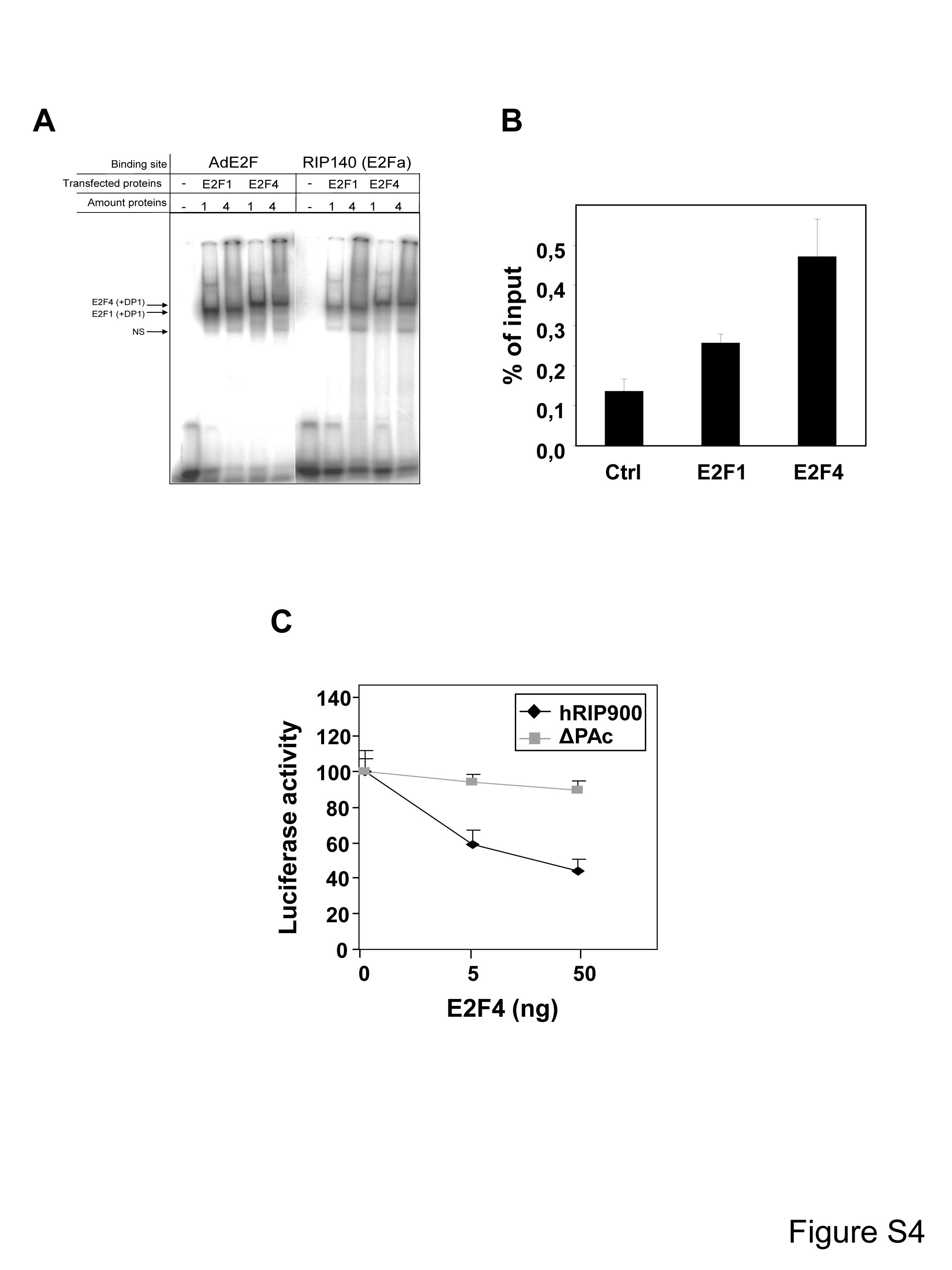

Supplement: Figure S4 — Binding of E2F4 on the distal site of the RIP140 promoter. (A) Electromobility shift assay was used to analyze E2F1/DP1 or E2F4/DP1 binding on the adenoviral E2F response element (Ad2E2F) or on the E2Fa site of the RIP140 promoter (see Figure 1). (B) ChIP experiments using immunoprecipitation (IP) of E21and E2F4 on the E2Fa site of the human RIP140 promoter. Data are expressed as percent of the PCR signal obtained with the amount of chromatin used for IP (input). Negative control of IP was done using an isotype-matched mAb. (C) MCF-7 cells were transiently transfected with the human and mutant ΔPAc RIP140 promoter reporter plasmids (25 ng) together with expression vectors for E2F1 and DP1 (25 ng each) and increasing amounts of E2F4 (0/5/50 ng). Relative luciferase activity was normalized with renilla luciferase activity as described in Materials and Methods, and is the mean (±SD) of triplicate. The values are expressed as a percentage of the activity obtained with control. (TIF) [file pone.0035839.s004.tif]

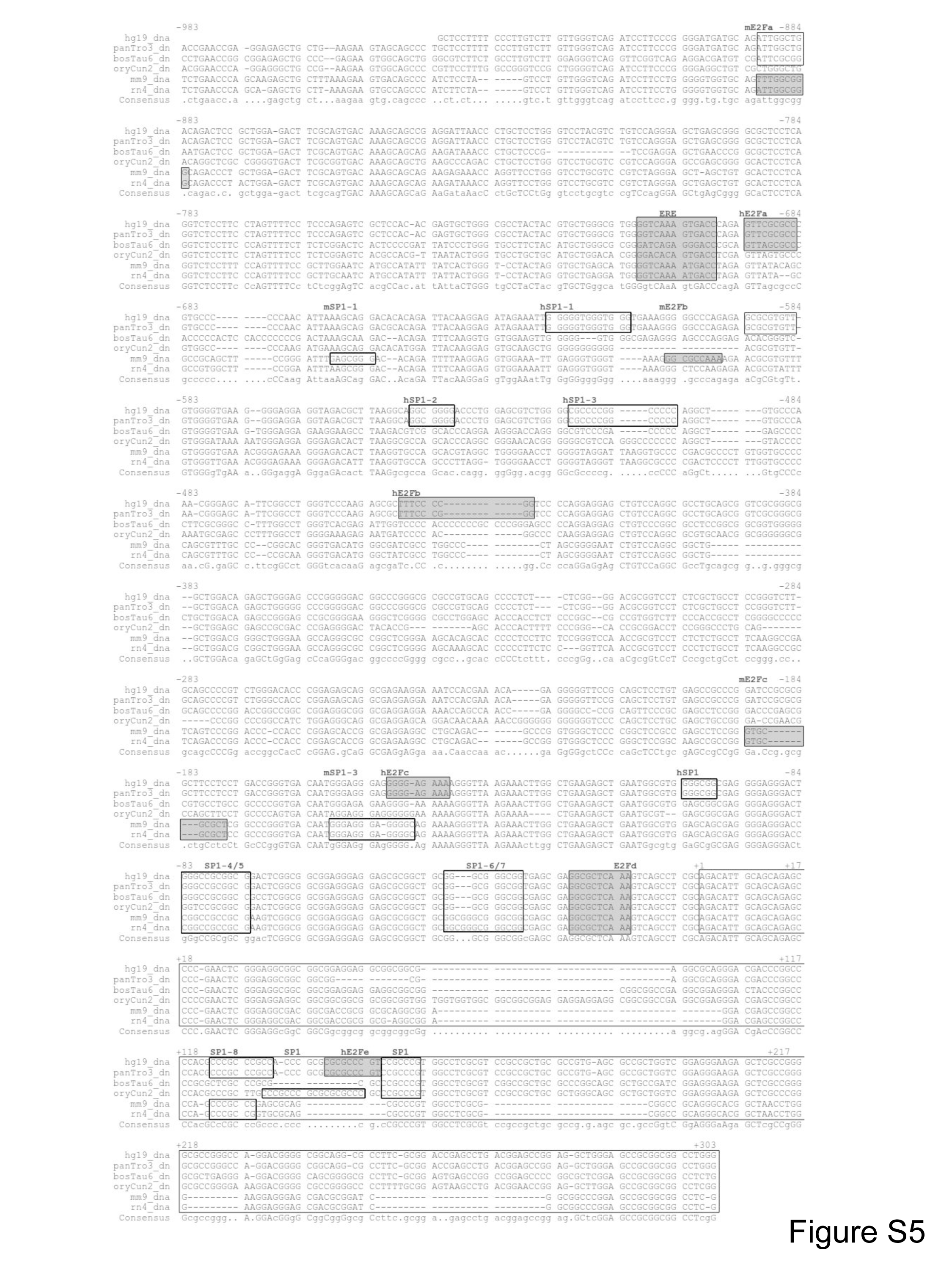

Supplement: Figure S5 — Conservation of the NRIP1 promoter sequence among mammals. The NRIP1 promoter regions of six mammalian species (from exon 1 b up to about 1 kbp) have been aligned using the Multalin program (hosted at http://multalin.toulouse.inra.fr/multalin/). Genome assembly used for each species were Homo sapiens (hg19), Pan troglodytes (panTro3), Bos taurus (bosTau6), Oryctolagus cuniculus (oryCun2), Mus musculus (mm9), and Ratus norvegicus (rn4) as indicated at the left of the aligned sequences that were extracted from the UCSC genome browser (http://genome.ucsc.edu/). Localization of putative E2F and Sp1 transcription binding sites was performed using MathInspector pattern search program from Genomatix (http://www.genomatix.de). E2F and Sp1 binding sites are shown in grey and white boxes respectively, with names above the sequence according to Figure 1 b and prefixed with h or m to distinguish human and mouse sites. The ERE that we [6] and others [7] found in both human and mouse promoters is shown as a landmark. Coordinates shown above the sequence are global to the alignment and are relative to the beginning of exon 1 b (large box at the end of the alignment). (TIF) [file pone.0035839.s005.tif]

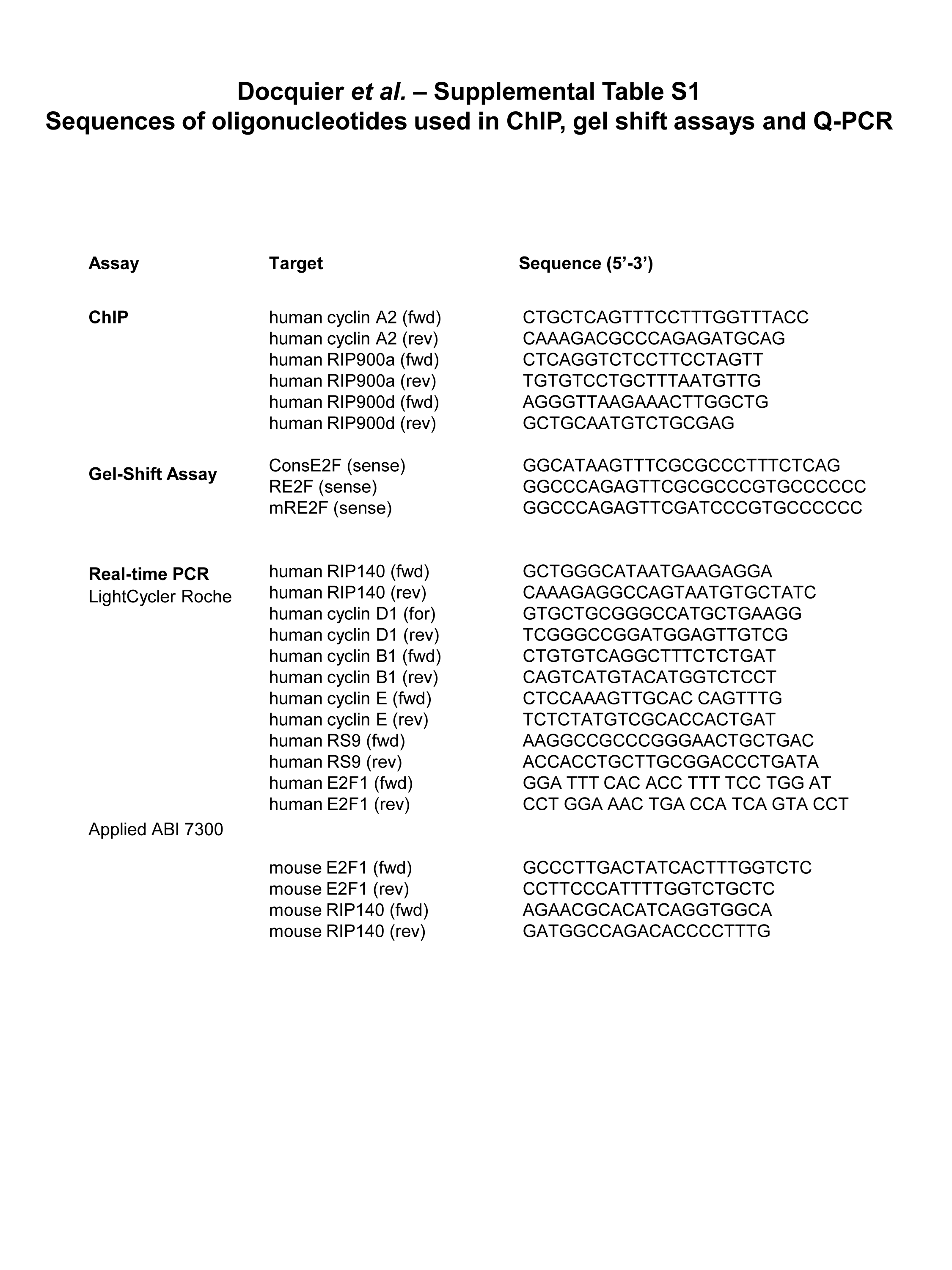

Supplement: Table S1 — Oligonucleotide sequences. The table shows the sequences of all the oligonucleotides used in the different assays (ChIP, gel shift assays and Q-PCR). The corresponding species (human or mouse) is indicated as well as the orientation of oligonucleotides i.e. sense/forward (fwd) or reverse (rev). The name of the target (promoter, binding site or mRNA) is also presented. (TIF) [file pone.0035839.s006.tif]
